# Differential levels of amino acid transporters System L and ASCT2, and the mTOR protein in placenta of preeclampsia and IUGR

**DOI:** 10.1186/1471-2393-14-181

**Published:** 2014-05-30

**Authors:** Yukiyo Aiko, David J Askew, Satoshi Aramaki, Mai Myoga, Chiharu Tomonaga, Toru Hachisuga, Reiko Suga, Toshihiro Kawamoto, Mayumi Tsuji, Eiji Shibata

**Affiliations:** 1 Department of Obstetrics and Gynecology, School of Medicine, University of Occupational and Environmental Health, Kitakyushu, Fukuoka, Japan; 2 Japan Environment and Children’s Study, UOEH Subunit Center, University of Occupational and Environmental Health, 1-1 Iseigaoka, Yahatanishi-ku, Kitakyushu, Fukuoka 807-8555, Japan; 3 Department of Environmental Health, School of Medicine, University of Occupational and Environmental Health, Kitakyushu, Fukuoka, Japan

**Keywords:** Placenta, Amino acid transporter, SLC3A2/4F2hc, SLC7A5/LAT1, SLC1A5/ASCT2, mTOR, Preeclampsia, Intrauterine growth restriction, Weight gain during pregnancy, Body-mass index

## Abstract

**Background:**

Sufficient amino acid transport activity (AAT) is indispensable for appropriate fetal growth. Studies suggest that placental nutrient uptake activity is responsive to both maternal and fetal nutrient demands. We hypothesize that under conditions of limited nutrient availability to the fetus, as often present in preeclampsia, intrauterine growth restriction (IUGR), and insufficient weight-gain during pregnancy, a general adaptive response aimed to increase amino acid transport activity may be observed in the placenta.

**Method:**

A total of 40 placentas from full-term (n = 10) and pre-term (average gestational period = 34.8 weeks, n = 10) normal pregnancies, IUGR (n = 10), and preeclampsia (n = 10) associated pregnancies were looked at by immunohistochemistry followed by relative qualitative scoring to compare expression levels and localization of System L, ASCT2, and mTOR proteins.

**Result:**

Microvillous syncytiotrophoblast (ST) in placenta of pregnancies complicated by IUGR or preeclampsia (PE) showed significant increases in the levels of System L amino acid transport proteins 4F2hc and LAT1 compared to both full-term control and pre-term (early gestation control) pregnancies seperately (p < 0.05). Elevated mTOR protein was uniquely higher in IUGR placentas compared to full-term controls (P = 0.0026). Total cellular ASCT2 transporter protein levels were similar in all groups, however, levels of ASCT2 protein localized to the ST microvillous membrane (MVM) were significantly lower in IUGR compared to both full-term and pre-term pregnancies (P = 0.0006, 0.03, respectively). Additionally, ASCT2 and mTOR protein levels were positively associated with maternal pre-pregnancy BMI (P = 0.046, 0.048, respectively).

**Conclusion:**

There are three important findings based upon the present study. First, in conditions of limited nutrient availability, such as PE or IUGR, there is an overall increase in the level of System L and mTOR protein expression in the ST, suggestive of an adaptive response. Second, a decrease in ASCT2 protein at the ST MVM suggests a post-translational event that may decrease AAT activity in IUGR placentas. Third, a physiological link between transporter expression and pre-pregnancy BMI is suggested based upon a positive association observed with ASCT2 and mTOR expression values.

## Background

Fetal growth restriction, as observed in intrauterine growth restriction (IUGR) and preeclampsia (PE), affects not only perinatal outcomes, but is also an important risk factor for developing diabetes, cardiovascular disease and other health problems in adulthood [1-3]. Nutrient availability and placental transport capacity are key determinants of fetal growth [4,5]. Amino acid transport in particular is intimately linked to fetal growth [6,7]. Lower activities for several of the amino acid transporter (AAT) systems have been documented in the placenta of IUGR fetuses including System A [8-10], System L [11], and taurine transport [12-14]. *In vivo* animal model studies also support the primary role of reduced amino acid transport activity in the development of IUGR [15,16]. The etiology of fetal growth restriction necessitates a better understanding of placental amino acid transport regulation.

Placental amino acid transport activity resides within the syncytiotrophoblast (ST) cells [17,18]. Efficient transport requires the coordination of both Na^+^-dependent and Na^+^-independent transporters. Sodium-dependent transporters, including System A (sodium-dependent neutral amino acid transporter 1 (SNAT1), −2, and −4/ SLC38A1,-2,-4) and System ASC (ASCT1/SLC1A4 and ASCT2/SLC1A5), are largely responsible for maintaining intracellular neutral amino acid substrate levels. The activity of System A in the microvillous membrane has been well described [8,10,19]. Na^+^-dependent ASCT2 expression has also been localized to placenta microvilli [20]. In normal tissues and cancer cells ASCT2 is critical to cell growth and survival as its glutamine transport activity supports amino acid exchangers including LAT1 [21-23]. However, there are no reports on ASCT2 activity relative to placenta function and fetal growth restriction. The sodium-independent transporters of System L (LAT1 and LAT2) exchange intracellular glutamine and other substrates for essential amino acids (EEAs) including Leucine and branched-chain amino acids (BCAA). LAT1 is expressed in the microvilli as a heterodimeric glycoprotein composed of the transporter-specific light chain LAT1/SLC7A5, and the common heavy chain 4F2hc/CD98/SLC3A2 [17,24]. The transport of branched-chain and EEAs has been shown to be affected in both IUGR (decreased) and LGA-associated placenta (increased) [19].

While the relationship between changes in amino acid transporter activities and pathological fetal growth is well established, their regulation is still poorly understood. The mammalian Target of Rapamycin (mTOR) protein appears to be a key component of AAT regulation [6,25,26]. mTOR is a Ser/Thr protein kinase which functions in diverse cell types, connecting growth factor signals with energy and nutrient levels, to control protein metabolism and cell growth [27]. In the placenta, mTOR has been shown to affect the activities of the System A, System L, and taurine AAT [25,28]. Further *in vitro* evidence ties mTOR activity to the sub-cellular localization of System A (SNAT2) and System L (LAT1) transporters [26].

Several lines of evidence support an adaptive model of fetal nutrient transport by which transporter function is altered based upon nutrient availability and fetal demand. Under limiting conditions, transport activity is increased in mice and trophoblast cell cultures [29,30]. Detailed analysis of tumor cells, in which amino acid transport activity and growth must also be adapted to fit limiting nutrient conditions, found that mTOR responses to amino acid concentrations are dependent on ASCT2 and LAT1 transporters, and their substrates L-Glutamine and Leucine, respectively [23,31,32]. The available evidence suggests that a similar system is present in the placenta ST, sensing fluctuations in nutrient availability and maintaining transport activities to achieve optimal fetal growth conditions [33]. These observations point to the importance of System L, ASCT2, and mTOR in the placenta as they pertain to fetal growth pathologies.

Preeclampsia and IUGR often arise from the common defect in placental development of impaired spiral artery remodeling [34,35]. This results in altered blood flow, and possible exposure of the developing fetus to a limited oxygen and nutrient supply. Pre-pregnancy BMI and inadequate maternal weight-gain during pregnancy may also result in restricted nutrient supply, and are additional risk factors for fetal growth restriction [36,37]. We hypothesized that in all conditions predicted to cause limited amino acid availability to the placental/fetal unit, similar adaptive responses aimed at increasing transport capacity, including increased AAT protein levels, may be observed. In the present study we investigated if the expression of 4F2hc, LAT1, ASCT2, and mTOR proteins in the placenta is changed in potentially nutrition-restricted conditions including preeclampsia and IUGR, or if their expression may be associated with maternal pre-pregnancy body mass index (BMI) or weight gain during pregnancy.

## Methods

### Study population and sample collection

Placental tissue collection was carried out with informed consent under the approval of the University of Occupational and Environmental Heath (UOEH) IRB Committee. Placentas were obtained primarily after caesarean delivery at full-term or pre-term from women with uncomplicated pregnancies giving birth to babies with normal birth weight (appropriate-for-gestational-age; AGA, full-term and pre-term control pregnancies), as well as from pregnancies complicated by IUGR or preeclampsia.

Preeclampsia (n = 10) was defined as gestational hypertension and proteinuria after 20 weeks gestation. Gestational hypertension was defined as new onset elevated maternal systolic blood pressure (BP) more than 140 mmHg or diastolic BP more than 90 mmHg. Proteinuria was defined as more than 300 mg protein in a 24 hour urine collection or more than 1+ on a catheterized urine specimen or more than 2+ on a voided specimen, or a random urinary protein/creatinine ratio of more than >0.3 [38]. IUGR (n = 10) was defined by a birth weight below the 10^th^ percentile (small for gestational age, SGA) in an otherwise uncomplicated pregnancy. Birth weight centiles were based upon Japanese gender-specific fetal growth data (adjusted for gestational age). Eight of ten IUGR babies exhibited asymmetric growth profiles. Asymmetric growth usually signifies IUGR-affected growth in the third trimester, resulting in a disproportionately low birth weight or length in comparison with occipital frontal head circumference. However, asymmetric growth has no universally accepted formula. Babies were considered to have asymmetric growth when the percentiles were disproportionate, generally following the pattern of total weight before liner growth before head circumference (*i.e.* weight centile < length centile < head centile). For these data, we defined “significantly less than” to mean plotting in nonadjacent percentile categories (<3rd, 3–5, 5–10, 10–25, 25–50, 50–75, 75–90, 90–95, 95–97, and > 97), wherein weight centile must be at least two categories below length and/or head circumference.

Table 1 shows the demographic data of case and control study subjects. Data are expressed as mean ± standard deviation (s.d.). The expected differences in blood pressure at delivery were observed among normal pregnancy, preeclampsia, and IUGR (systolic/diastolic: 121 ± 9/73 ± 9 mmHg, 162 ± 19/97 ± 10 mmHg, and 122 ± 24/77 ± 22 mmHg), and all were normotensive pregnancy before 20 weeks gestation. Gestational weeks at delivery of pre-term controls, preeclampsia and IUGR were about 5 weeks earlier compared to uncomplicated full term pregnancies. Infant birth weight centile was significantly lower in preeclampsia or IUGR compared to uncomplicated full term and preterm pregnancies. The incidence of maternal smoking was not different between groups. Caesarean section in uncomplicated pregnancy was due to repeat-caesarean section or breech presentation. A limited number of samples were obtained after vaginal delivery, particularly in the pre-term normal pregnancy group due to the limited number of available samples.

**Table 1:** Characteristic averages of pregnancy groups

	**Full term control**	**Preterm control**	**Preeclampsia**	**IUGR**
Maternal age	31.0 ± 6.4	31.0 ± 4.9	34.0 ± 4.3	31.6 ± 6.0
Maternal BMI (kg/m2)	21.3 ± 6.6	20.0 ± 3.7	23.8 ± 5.0	17.8 ± 1.2
Percent nulliparous	70	30	30	70
Blood pressure at delivery (mmHg)				
Systolic (mmHg)	121 ± 9	116 ± 7	162 ± 19 ^a^	122 ± 24
Diastolic (mmHg)	73 ± 9	66 ± 10	97 ± 10^c^	77 ± 22^d^
Blood pressure <20 weeks GA				
Systolic (mmHg)	118 ± 7	109 ± 6	141 ± 11	105 ± 21
Diastolic (mmHg)	77 ± 11	59 ± 10	87 ± 6	64 ± 12
Gestational weeks at delivery	39.2 ± 1.5	34.4 ± 0.7	33.6 ± 2.0^a^	34.5 ± 3.1^a^
Birth weight (g)	2835 ± 435	1993 ± 236	1446 ± 333^a^	1465 ± 335^a^
Birth weight centile	35.8 ± 25.1	30.4 ± 19.8	5.2 ± 7.3^a^	2.6 ± 3.6^a^
Placental weight (g)	558 ± 141	448 ± 122	348 ± 91^b^	332 ± 82^a^
Smoking (%)	0	10	0	10
Ceasarean delivery (%)	90	30	80	60

Continuous variables are shown as mean ± s.d. Dichotomous variables are given as a percentage. Smoking status was self-reported. BMI, body mass index; BP, blood pressure; *n* =10 samples/case condition.

^a^*P value < 0.0001* (Full term control vs Preeclampsia, IUGR); ^b^*P value 0.0003* (Full term control vs Preeclampsia, IUGR); ^c^*P value 0.0009* (Full term control vs Preeclampsia); ^d^*P value 0.0055* (Preeclampsia vs IUGR).

The clinical characteristics for each newborn with IUGR or born to women with preeclampsia is presented in Table 2. Newborns 1 to 10 are normotensive IUGR-associated, and 11 to 20 are associated with preeclampsia. All but two infants (number 12 and 14) born to women with preeclampsia also fit the criteria of SGA. The rate of oligohydramnios was 30% in both preeclampsia and IUGR. The infant of patient number 5 in Table 2 exemplifies asymmetric growth. This infant has a birth weight centile of 1.4 (category < 3rd), length percentile < 3, and head circumference centile 10–25 (two categories greater than birth weight and length). Eight of ten IUGR babies, and six of the eight SGA babies in preeclampsia, exhibited asymmetric profiles.

**Table 2:** Clinical characteristics of IUGR and preeclampsia

**Patient #**	**Oligo-hydramnios**	**Birth weight centile**	**Length centile**	**Head circumference centile**	**Growth profile**
**1**	**-**	0.3	0.0	10.1	Asymmetrical
**2**	**-**	9.3	3.8	41.2	Asymmetrical
**3**	**-**	9.2	42.0	78.0	Asymmetrical
**4**	**+**	1.3	2.3	19.3	Asymmetrical
**5**	**-**	1.4	2.4	11.0	Asymmetrical
**6**	**-**	1.4	13.9	12.5	Asymmetrical
**7**	**-**	0.0	0.6	0.6	Symmetrical
**8**	**-**	3.8	6.0	28.3	Asymmetrical
**9**	**+**	0.0	0.1	0.8	Symmetrical
**10**	**+**	0.0	0.0	8.2	Asymmetrical
**11**	**+**	7.1	14.0	35.1	Asymmetrical
**12**	**-**	16.1	65.2	33.8	Symmetrical
**13**	**-**	0.4	0.0	21.1	Asymmetrical
**14**	**-**	20.7	13.1	55.7	Asymmetrical
**15**	**+**	1.7	18.4	55.9	Asymmetrical
**16**	**-**	0.6	1.8	12.3	Asymmetrical
**17**	**-**	4.2	10.5	0.1	Symmetrical
**18**	**-**	0.6	8.2	3.1	Symmetrical
**19**	**-**	0.2	1.3	0.5	Symmetrical
**20**	**+**	0.4	0.0	19.1	Asymmetrical

Clinical data of IUGR (no.1-10) and Preeclampsia (no.11-20) pregnancies are shown above.

**+**, positive for oligohydramnions; − , negative for oligohydramnions.

Birth weight centile is listed as a continuous variable. Asymmetric growth was determined by comparison of birth weight, length, and head circumference centiles for each neonate as described in Methods.

### Immunohistochemistry

Formalin fixed paraffin embedded placental tissue samples (1 sample per placenta, collected between the rim and point of chord insertion) were obtained from the tissue bank facility of the UOEH Pathology Department with IRB approval (full term pregnancy, n = 10; pre-term, n = 10; preeclampsia, n = 10; and IUGR, n = 10). Paraffin sections (3.5 μm) were incubated overnight at 37°C or 1 hr at 65°C, deparaffinized in xylenes and rehydrated in ethanol and water. Slides underwent antigen retrieval in citrate buffer, pH6.0, followed by peroxidase blocking (Block, Dako, Tokyo, Japan) before incubation with the appropriate primary Ab diluted in PBS, 1 hr, RT. Detection was performed using Envision HRP-conjugated secondary Ab and DAB color development system (DAKO) for consistent development time between samples. Immunohistochemistry (IHC) results were qualitatively estimated in a blinded fashion by two individuals. Chromogenic signal intensity was assigned a relative score of 0 (no signal), 1 (weak signal detected), 2 (moderate), and 3 (strong) [39]. The scores of five fields of view were averaged for each slide. For each placenta sample and each antigen, two or three slides made from non-consecutive sections were stained and scored in independent IHC experiments.

### Antibodies and chemicals

Primary antibodies recognized Cytokeratin7 (Sigma, St. Louis, MO, USA); 4F2hc and LAT1 (KEO20 and KEO23, respectively, Transgenic, Inc., Kobe, Japan); mTOR (ab2732, Abcam, Tokyo, Japan); and ASCT2/SLC1A5 (H-52, Santa Crus, CA, USA). All chemicals were purchased from Sigma unless noted.

### Data presentation and statistics

The sample size (*n*) is the number of different placentas representing each case or control group. Clinical characteristics data (Tables 1 and 2) were assessed by ANOVA. IHC scoring was assessed by t-test. Statistical significance was accepted at p < 0.05.

## Results

### Comparison of AAT protein expression levels between PE, IUGR and both full- and pre-term uncomplicated pregnancies using Immunohistochemistry

**Figure 1:**
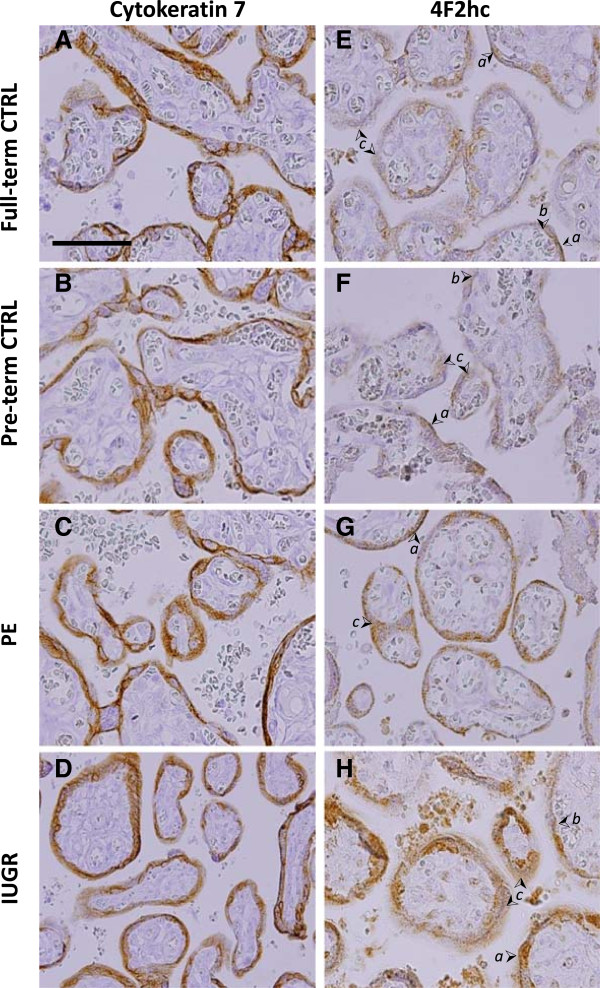
**Localization of Cytokeratin and the AAT 4F2hc.** Immunohistochemistry staining for Cytokeratin 7 **(A-D)** and AAT 4F2hc **(E-H)** in placenta from Full-term control (CTRL) **(A,E)**, Preterm control **(B,F)**, PE **(C,G)**, and IUGR, **(D,H)** associated placentas. (➢ Arrow denotes syncytiotrophoblast apical MVM (*a*), basal membrane *(b)*, and cytoplasm (*c)*, or cytotrophoblasts (*ct*). Size bar in A = 100 μm).

**Figure 2:**
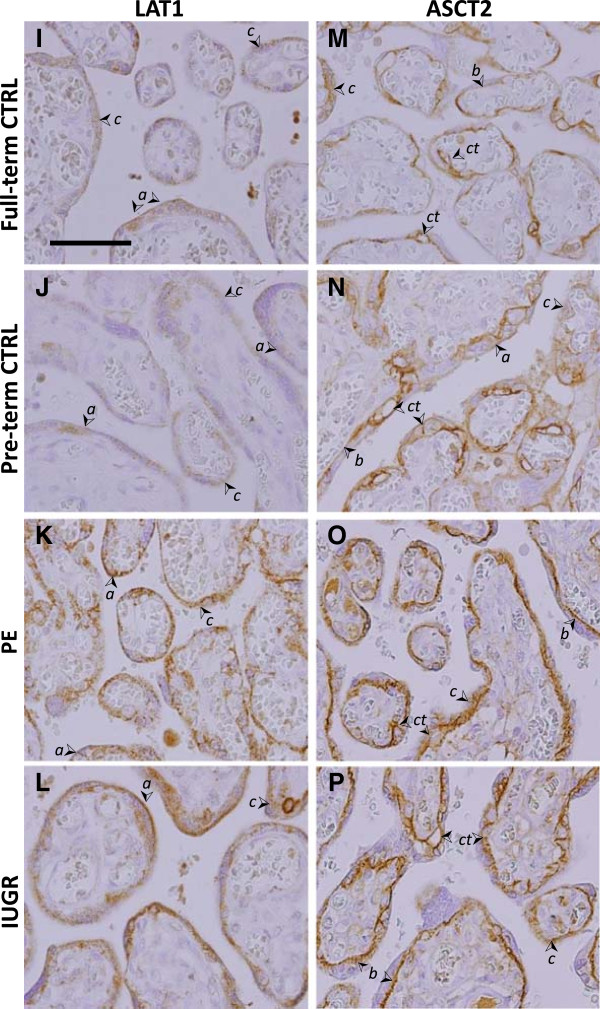
**Localization of the AATs LAT1 and ASCT2.** Immunohistochemistry staining for LAT1 **(I-L)** and ASCT2 **(M-P)**, in placenta from Full-term control (CTRL) **(I,M)**, Preterm control **(J,N)**, PE **(K,O)**, and IUGR **(L,P)** associated placentas. (➢ Arrow denotes syncytiotrophoblast apical MVM (*a*), basal membrane *(b)*, and cytoplasm (*c)*, or cytotrophoblasts (*ct*). Size bar in A = 100 μm).

To begin investigating the regulation of AAT under conditions of normal and limiting nutrient availability, the expression level and localization of three AAT proteins, LAT1, 4F2hc, and ASCT2, were determined in placenta samples from IUGR, PE and both full-term and maternal age-matched pre-term controls using immunohistochemistry (IHC). Cytokeratin 7 (CK7) was included as a ST cell marker, and as a control for levels of non-nutrient transport-associated proteins. CK7 was present predominantly throughout the ST of the microvillous. CK7 levels were similar between all four pregnancy groups (Figure 1, A-D). AAT proteins 4F2hc, LAT1, and ASCT2 were also localized to the ST of the microvillous (Figures 1, E-H, and 2, I-P).

The common heavy chain subunit 4F2hc was primarily observed in microvillous ST, as previously described [24,40]. Staining was diffuse across the cytoplasm. Some localized signal was present at both the apical MVM and basal plasma membranes (Figure 1, E-H). The light chain subunit LAT1 was also observed in the ST, with additional staining in some stromal cells of the microvillous. (Figure 2, I-L). LAT1 was partially concentrated at the apical MVM, and was detected throughout the cytoplasmic compartment. Subcellular localization of 4F2hc and LAT1 was similar in full-term and pre-term controls and both PE and IUGR groups. Interestingly, 4F2hc and LAT1, staining was of greater intensity in IUGR and PE associated placenta compared to both the full-term and pre-term controls (Figures 1, E-H, and 2, I-L).

**Figure 3:**
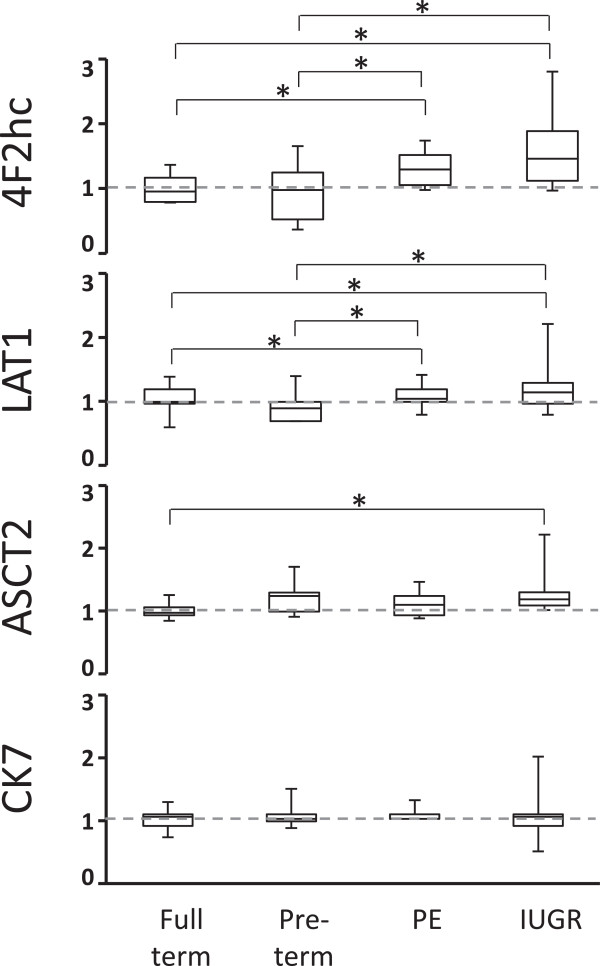
**Amino Acid Transporter protein levels in placenta microvillous syncytiotrophoblasts.** Protein levels were estimated by blind scoring of IHC-stained formaldehyde fixed samples from the 4 different pregnancy conditions (n = 10 each, * p < 0.05). Data is presented in box and whisker format to define minimum and maximun values (capped bars), 2^nd^ and 3^rd^ quartile (box top and bottom) and median (line through box). Data was normalized to full term controls (mean set to 1, grey dashed line).

To quantify these differences in AAT protein levels, blinded scoring of the IHC results was performed. Multiple fields of view from each stained slide were assigned a relative score of 0 (no signal), 1 (weak signal detected), 2 (moderate), and 3 (strong) based upon staining intensity [39]. Scoring of signal intensity confirmed that 4F2hc and LAT1 protein levels were significantly higher in both PE and IUGR, compared to controls (p < 0.05, Figure 3). No significant differences were measured between full-term and pre-term control groups, or between PE and IUGR, although 4F2hc levels in IUGR scored the highest. For comparison, CK7-specific staining intensity was scored and no difference in intensity between groups was observed (Figures 1, A-D, and 3).ASCT2 protein was localized to both the microvillous ST and cytotrophoblasts (CT) by IHC (Figure 2, M-P). Staining was strongest in CT membranes and the ST basal plasma membrane. Some variation was observed in staining intensity in CT between samples. In some samples, staining was very strong around the entire CT membrane perimeter, while staining was limited to regions bordering with the ST in others. Overall, we were unable to detect any association of these distinct staining patterns with samples from either uncomplicated or pathological conditions. Estimation of ASCT2 protein levels using the blinded scoring method found the only significant difference in ASCT2 to be between the IUGR and full-term control groups (Figure 3).

**Figure 4:**
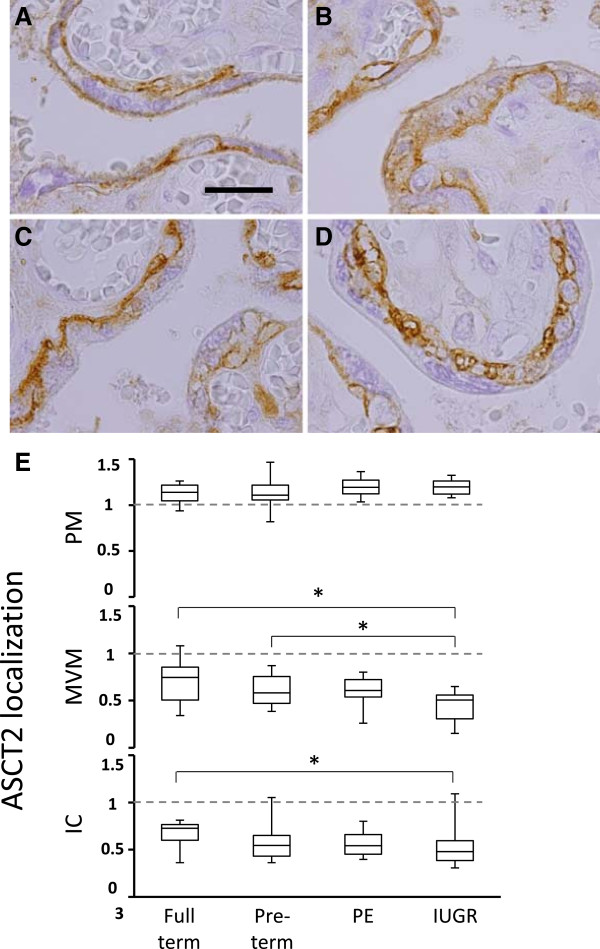
**ASCT2 protein localization within the syncytiotrophoblast.** ASCT2 protein was detected by immunohistochemistry in the placenta of **A**, full-term control, **B**, pre-term control, **C**, preeclampsia and **D**, intrauterine growth restriction-associated pregnancies. **E**, ASCT2 protein expression levels were estimated in the syncytiotrophoblast, including subcellular localization at the plasma membrane (PM), microvillus membrane (MVM), and the intracellular compartment (IC) (* P < 0.05, size bar = 40 mM).

In addition to overall protein levels within the ST layer, we also wished to compare AAT protein localization, a common mechanism of AAT activity regulation [30,41]. By IHC, both 4F2hc and LAT1 appeared relatively diffuse, with proteins being detected in ST cytoplasm and MVM, making a quantitative estimation with respect to localization very difficult. ASCT2 exhibited strong staining of the cytotrophoblast as well as ST basal plasma membrane, and this staining appeared consistent across all samples. However, different patterns of staining with respect to ST intracellular cytoclasmic space and MVM localization were present within the sample set (Figure 4, A-D). The same blinded scoring system was employed to grade ST cytoplasmic and MVM localized staining intensity for ASCT2. Pre-term control and PE placenta samples both exhibited reduced levels of ASCT2 at the MVM and in the intracellular compartment compared to full-term controls, but these differences were non-significant. The IUGR group exhibited the lowest ASCT2 levels at the MVM (ASCT2_MVM_) and in the intracellular compartment (ASCT2_IC_) (Figure 4). IUGR ASCT2_MVM_ levels were significantly different from both full-term and pre-term control groups (P = 0.0006, 0.017, respectively), and IUGR ASCT2_IC_ was significantly lower than the full-term control group (P = 0.03, Figure 4E).

### Comparison of mTOR protein levels between Normal, PE and IUGR-associated placenta

**Figure 5:**
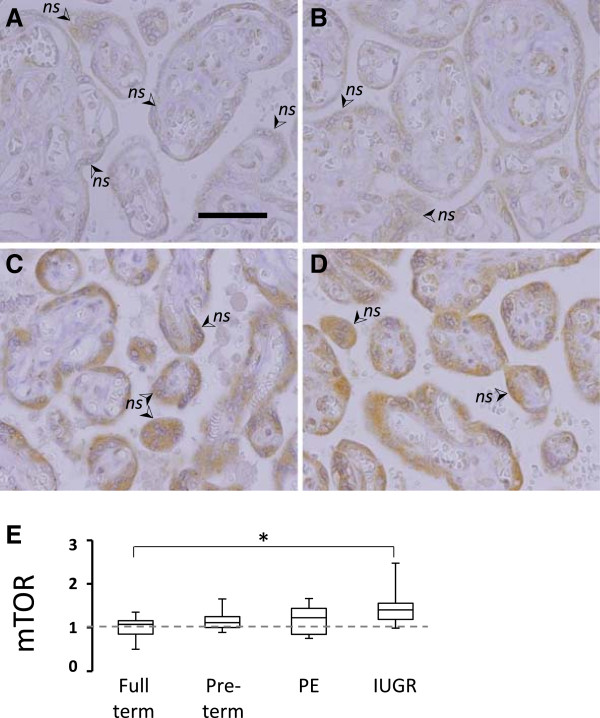
**mTOR protein expression in placenta ST.** Placenta of **A**, full-term control, **B**, pre-term control, **C**, preeclampsia, and **D**, intrauterine growth restriction-associated pregnancies were probed with antibodies recognizing total mTOR protein. (➢ Arrow denotes *ns*, nuclei syncytia. Size bar in A = 100 mM). **E**, Estimated mTOR IHC scoring data is presented in box and whiskar format to define minimum and maximun values (capped bars), 2^nd^ and 3^rd^ quartile (box) and median (line through box). Data was normalized to full term controls (mean set to 1, grey dashed line) (n = 10 each, * p < 0.05).

Our current understanding of amino acid transporter regulation includes a central role for mTOR in responding to nutrient levels and hormone stimuli. Therefore we continued our immunohistological investigation of predicted differences in AAT machinery with the examination of mTOR in these four placenta groupings. The cellular localization of mTOR in term, pre-term, PE, and IUGR pregnancies appeared similar, primarily cytoplasmic in the ST (Figure 5, A-D). Stronger staining, corresponding to higher mTOR protein levels, was observed in all three groups associated with early gestation periods, compared to full-term pregnancy. mTOR staining in IUGR associated samples was the strongest, and significantly different from full-term controls (p = 0.002, Figure 5, A-D) but not from pre-term controls (p = 0.07). In both PE and IUGR samples, intense mTOR staining was frequently co-localized with nuclear syncytia (Figure 5, C and D).

### Examination of AAT and mTOR protein levels in relation to weight gain during pregnancy and pre-pregnancy BMI

To further test the hypothesis that limited nutrient availability may stimulate compensatory increases in AAT expression in order to increase transport potential, we examined the relationship between AAT and mTOR protein expression with weight gain during pregnancy and pre-pregnancy BMI, factors directly associated with placental nutrient availability and fetal growth [36,37,42]. To do this, the protein expression levels estimated by blinded scoring of IHC results for the 40 samples representing full- and pre-term pregnancies, PE and IUGR-associated pregnancies, were analyzed with respect to weight gain and BMI (Table 3). First, AAT and mTOR levels were compared between two weight gain groups, less than adequate- and equal to or greater than adequate weight gain/week. Adequate weight gain was defined according to the 2009 Institute of Medicine guidelines which take into account pre-pregnancy BMI values in estimating adequate weight-gain ranges [43]. Briefly, adequate weight gain ranges for different BMI values were as follows: BMI < 18.5 kg/m2, 0.44-0.58 kg/week; BMI of 18.5 to 24.9 kg/m2, 0.35-0.5 kg/week; BMI of 25 to 29.9 kg/m2, 0.23-0.33 kg/week; and BMI > 29.9 kg/m2, 0.17-0.27 kg/week.

**Table 3:** Association of AA Transporter, mTOR expression with Maternal Weight Gain and BMI

**Protein/cellular localization**^**a**^	**Weight gain/week of pregnancy**				**Pre-pregnancy BMI**			
	**< adequate**	**= or > adequate**	**P value**	**Trend**^**c **^**(< → =/>)**	**Low**	**High**	**P value**	**Trend**^**c **^**(low → high)**
4F2hc	1.26 ± 0.51	1.03 ± 0.28	0.211	↓	1.11 ± 0.33	1.29 ± 0.59	0.22	↑
LAT1	1.07 ± 0.25	1.10 ± 0.27	0.791	nc	1.08 ± 0.25	1.09 ± 0.27	0.93	nc
ASCT2	1.12 ± 0.16	1.18 ± 0.14	0.855	nc	1.07 ± 0.15	1.17 ± 0.16	0.046*	↑
mTOR	1.18 ± 0.27	1.12 ± 0.27	0.537	nc	1.08 ± 0.31	1.26 ± 0.24	0.048*	↑
mvmASCT2^b^	0.58 ± 0.19	0.58 ± 0.18	0.991	nc	0.64 ± 0.20	0.53 ± 0.18	0.107^c^	↓

*Significant differences (p < 0.05) between low and high BMI groups (lower and upper 50%) was estimated by t-test.

^a^Protein expression is the average of individual sample values, as described in Methods, sorted into each weight gain or BMI grouping (n = 15 for each).

^b^mvmASCT2 refers to ASCT2 protein levels localized to the syncytiotrophoblast microvillous membrane.

^c^Trend refers to an observed increase (↑), decrease (↓), or no change (nc) in protein expression between the weight gain or BMI groupings.

Elevated placenta levels of 4F2hc were observed in the less than adequate weight gain group relative to the adequate and above weight gain group, although this difference was not found to be significant by t-test (1.26 ± .51 vs. 1.03 ± .28 respectively; p = 0.211). No difference between weight gain groups was observed in over-all staining levels for any of the AAT or mTOR proteins, or ASCT2 subcellular localization (Table 3). Second, AAT and mTOR protein level values were sorted equally (n = 20 for each) into lower (BMI range 15.7 to 17.4) and upper pre-pregnancy BMI groups (BMI range 17.7 to 35). In this case, significant associations were identified. Elevated ASCT2 and mTOR protein levels were associated with higher BMI values (p = 0.046, 0.048, respectiviely; Table 3). Overall, these observations further help to define the extent of amino acid transporter regulation in the placenta and its close association with both nutrient availability and maternal metabolism as it relates to pre-pregnancy BMI.

## Discussion

### Summary

Amino acid transporter and mTOR protein levels in placental syncytiotrophoblast cells were estimated by immunohistochemistry in a blinded and relative qualitative scoring system [39]. Our findings support the adaptive response hypothesis of placental nutrient transport function, and suggest that AAT protein levels and cellular localization are affected by limiting nutrient availability. Specifically, increases in protein levels of System L amino acid transporter LAT1 and regulator mTOR, as well as changes in subcellular localization of ASCT2 protein were observed in PE and IUGR, compared to full- and pre-term normal pregnancy controls.

### The importance of AAT and the adaptive response

Fetal growth is dependent on maternal nutrient supply and placenta nutrient transport capacity. The latter is determined by the expression and regulation of multiple transport systems. Several lines of evidence confirm that reduced amino acid transport capacity significantly contributes to the IUGR phenotype including lower placental AAT activities, and protein levels in isolated syncytiotrophoblast membranes [8,11-14,19]. However, the mechanism by which placental AAT activity may be reduced so severely as to promote IUGR it is not known. The experiments described here were aimed at investigating AAT protein levels *in situ* and associations during normal and restricted growth conditions. Our observations of elevated 4F2hc and LAT1 protein in ST of PE and IUGR placenta may be unexpected and potentially conflicting with the reports described above of both reduced AAT activities and protein levels in isolated microvillous membranes. However, these data do support the involvement of an adaptive response in placentas associated with both PE and IUGR in the form of increased transporter protein expression. In the adaptive response model nutrient supply and fetal demand are sensed by the placenta and adjustments are made to maintain an adequate nutrient supply [34]. This adaptive response is well described in muscle believed to be important in the placenta for regulation of System L, as well as System A and other transporter systems [6,30,41,44].

Similar changes in AAT protein expression were observed in PE as in IUGR, which we argue is because the PE group is also associated with conditions of reduced blood flow and nutrient restriction. PE is currently described as a disease with two distinct contributing sets of factors, insufficient placentation (placental preeclampsia), and maternal deficiencies of the endothelium and other systems (maternal preeclampsia) [35]. Staff, et al., state that all PE cases are likely due to a combination of both maternal and placental factors. Our PE sample set (n = 10) included 8 SGA births, and two births below the 20^th^ centile gestational weight at birth. Therefore, while nutrient insufficiency may not be as severe in some IUGR cases, placental insufficiency and limiting nutrient availability is likely a major component of all PE cases included in this study.

### mTOR protein levels in IUGR verses PE

In addition to increases in AAT protein levels, we also observed an increase in total mTOR protein levels in IUGR-associated placenta. Unlike the AAT proteins, the mTOR increase was unique to IUGR, not being observed in the PE group. Recent work has identified the protein kinase mTOR as an important component of the signaling pathway between nutrient availability, insulin hormone signaling, and AAT activity in the placenta [28,45]. Our work is in agreement with Roos, et al., who observed increased total mTOR protein in IUGR [25]. Here, we extend these observations by demonstrating a link between mTOR and pre-pregnancy BMI values as well. In cancer cells, increased mTOR signaling is believed to allow survival under nutrient-restricted conditions of the tumor environment [46]. Our observation is limited to total mTOR protein levels, as it does not include an analysis of relevant mTOR kinase substrates or the phosphorylation state of mTOR itself. Additional work is needed to evaluate mTOR activities in the human placenta *in vivo*, comparing normal and pathological conditions.

### Post-translational control of nutrient transporters

We observed a loss of ASCT2 protein in the MVM and intracellular compartment in IUGR-associated placenta. This may directly result in the reduced protein transport activities expected to be so critical in the development of IUGR. ASCT2 function may be particularly important because of its role in maintaining intracellular levels of glutamine, the main substrate for amino acid exchangers including System L. The importance of ASCT2 and glutamine transport has recently come to light with reports that elevated ASCT2 activity allows many cancers to escape the restriction of limited nutrient availability by increasing overall amino acid transport [47]. Knocking down ASCT2 protein expression *in vitro* confirmed that its glutamine transport function was a rate limiting step for other amino acid transport activities.

Our observation lends additional support to the importance of post-translational regulation of nutrient transport activities in the placenta. Reports by Roos et.al, and Rosario et al. demonstrate the important role of post translational regulation via protein trafficking in syncytiotrphoblast cultures [26,45]. In the first, mTOR inhibition was shown to reduce AAT activity [45]. More recently, inhibition of mTOR was shown to modify the post translation regulation of AAT, resulting in decreased plasma membrane localization [26]. Based upon the available data, one model predicts low mTOR activity would be dominant to the presence of a compensatory increase of AAT protein expression, resulting in reduced AAT transport activity in the syncytiotroblast.

### Maternal weight gain, BMI and nutrient restriction

Pre-pregnancy body-mass index (BMI) and maternal weight gain during pregnancy has a strong influence over fetal weight at gestation. Insufficient weight gain is associated with an increased risk of IUGR, preterm birth, and perinatal mortality, while sufficient weight gain may overcome additional risks for restricted fetal growth [36,37,42]. In 2009, the Institute of Medicine (IOM) of the National Academy of Sciences reexamined the guidelines for weight gain during pregnancy, and utilized BMI values to established optimal ranges of weight gain for the best maternal and fetal outcomes [43]. A recent survey demonstrated the importance of BMI values and appropriate nutritional availability to the fetus based upon these guidelines [48]. Taking advantage of the predicted differences in nutrient availability to the fetus associated with BMI and changes in gestational weight gain, we examined AAT and mTOR levels with respect to both gestational weight gain and pre-pregnancy BMI, independently. Interestingly, maternal pre-pregnancy BMI, but not weight gain, was estimated to be a predictor of both ASCT2 and mTOR levels in placenta, with higher levels of both proteins associated with higher BMI values. This result for ASCT2 parallels the positive association of BMI and reduced risk of fetal growth restriction [37,43]. It also provides additional evidence in support of the importance of ASCT2 and mTOR activity in the placenta.

## Conclusions

In IUGR and preeclampsia, the fetus may be exposed to nutrient limitations caused by abnormal remodeling of the uterine spiral arteries [49]. However, a successful adaptive response is predicted to result in sufficient placental nutrient transport to overcome a state of fetal growth restriction. Our data may be explained by a model in which two events regulate AAT activity. First, an adaptive response includes increased System L transporter protein expression in ST, as observed here in both PE and IUGR. Second, post-translational modifications altering AAT protein localization including ASCT2 may lead to the loss of microvillous membrane localized AAT, and therefore an overall loss of activity, resulting in growth-limiting levels of amino acid transporter activity.

## Abbreviations

AAT: Amino acid transporter; ASCT2: Alanine/ Serine/ Cysteine/ Threonine Transporter 2; mTOR: Mammalian target of Rapamycin; SLC3A2: Solute carrier family 3 member A2; SLC38A1: Solute carrier family 8 member A1; SLC1A4: Solute carrier family 1 member A4; SLC5A7: Solute carrier family 5 member A7; 4F2hc: Amino acid transporter protein 4 F2 heavy chain; LAT1: L-type amino acid transporter; EEA: Essential amino acids; BCAA: Branched-chain amino acids; PE: Preeclampsia; IUGR: Intrauterine growth restriction; AGA: Appropriate for gestational age; SGA: Small for gestational age; BMI: Body mass index; BP: Blood pressure; mmHg: millimeters of Mercury; ST: Syncytiotrophoblast; CT: Cytotrophoblast; MVM: Microvillous membrane; SNAT: Sodium-dependent neutral amino acid transporter; s.d.: Standard deviation; PBS: Phosphate buffered saline; Ab: Antibody; IHC: Immunohistochemistry; HRP: Horse radish peroxidase; DAB: 3,3*'* Diaminobenzidine; ANOVA: Analysis of variance; CK7: Cytokeratin 7; IC: Intracellular; IOM: Institute of Medicine.

## Competing interests

The authors have no competing interests to declare.

## Author’s contributions

YA carried out immunohistochemistry experiments, blinded scoring, and statistical analysis, and participated in data interpretation, generating figures, writing and revising. DJA carried out immunohistochemistry experiments and blinded scoring, participated in data interpretation, generating figures, writing and revising. SA, MM and CT participated in sample collections and immunohistochemistry experiments. TH acquired funding and participated in experiment design, data interpretation, writing and revising. RS participated in analyzing and interpreting data, generating figures, writing and revising. TK acquired funding and participated in experiment design and data interpretation. MT participated in experiment design and data interpretation, writing and revising. ES acquired funding and participated in experiment design and data interpretation, writing and revising. All authors read and approved the final manuscript.

## Pre-publication history

The pre-publication history for this paper can be accessed here:

http://www.biomedcentral.com/1471-2393/14/181/prepub
